# Is Bhutan destined for 100% organic? Assessing the economy-wide effects of a large-scale conversion policy

**DOI:** 10.1371/journal.pone.0199025

**Published:** 2018-06-13

**Authors:** Arndt Feuerbacher, Jonas Luckmann, Ole Boysen, Sabine Zikeli, Harald Grethe

**Affiliations:** 1 International Agricultural Trade and Development Group, Humboldt-Universität zu Berlin, Berlin, Germany; 2 School of Agriculture and Food Science, University College Dublin, Dublin, Ireland; 3 Institute of Crop Science, Coordination for Organic Farming and Consumer Protection, University of Hohenheim, Stuttgart, Germany; California State University Fresno, UNITED STATES

## Abstract

Organic agriculture (OA) is considered a strategy to make agriculture more sustainable. Bhutan has embraced the ambitious goal of becoming the world’s first 100% organic nation. By analysing recent on-farm data in Bhutan, we found organic crop yields on average to be 24% lower than conventional yields. Based on these yield gaps, we assess the effects of the 100% organic conversion policy by employing an economy-wide computable general equilibrium (CGE) model with detailed representation of Bhutan’s agricultural sector incorporating agroecological zones, crop nutrients, and field operations. Despite a low dependency on agrochemicals from the onset of this initiative, we find a considerable reduction in Bhutan’s GDP, substantial welfare losses, particularly for non-agricultural households, and adverse impacts on food security. The yield gap is the main driver for a strong decline in domestic agricultural production, which is largely compensated by increased food imports, resulting in a weakening of the country’s cereal self-sufficiency. Current organic by default farming practices in Bhutan are still underdeveloped and do not apply the systems approach of organic farming as defined in the IFOAM organic farming standards. This is reflected in the strong decline of nitrogen (N) availability to crops in our simulation and bears potential for increased yields in OA. Improvement of soil-fertility practices, e.g., the adoption of N-fixing crops, improved animal husbandry systems with increased provision of animal manure and access to markets with price premium for organic products could help to lower the economic cost of the large-scale conversion.

## Introduction

There is an uncontested need to reduce the environmental impacts of agriculture. Converting from a conventional to an organic agricultural system is viewed as one strategy to meet this need [1]. A key challenge is, however, not to compromise the supply of food and biomass. Current studies on large-scale conversions vigorously debate the existence and size of yield gaps between conventional and organic agriculture [2] and the question of whether 100% organic agriculture (OA) can feed a growing world population [3–6]. So far, little research has been conducted on the economy-wide impacts of large-scale conversions to OA on a national scale. This might be due to the lack of genuinely formulated policy objectives, which aim to convert the entire agricultural sector on a large scale. Bhutan has received remarkable international attention following its announcement to become the world’s first 100% organic nation by 2020 [7].Besides environmental conservation, which is one of the four pillars of Bhutan’s Gross National Happiness philosophy, there are also other motifs for this policy, such as promoting the brand “Bhutan” [8].

OA is a holistic agricultural system that strictly relies on natural inputs and promotes practices like the maintenance of soil fertility, conservation of biodiversity, and animal welfare according to the norms of the International Federation of Organic Agriculture Movements (IFOAM) [9]. The usage of most chemical fertilizers (except for mineral phosphate and potassium not enriched by chemical processes), pesticides and genetically modified crops is prohibited. In animal husbandry, the use of artificial growth hormones is banned, and antibiotics are only allowed in exceptional cases. In 2012, 37% of Bhutan’s farmers used agrochemicals on about 19% of arable land [10]. Given its low reliance on agrochemicals, there is the notion that the country is destined for 100% OA [11]. However, this notion does not properly account for the difference between “organic by default” (i.e., no use of chemical inputs and mineral fertilisers) and organic by IFOAM principles, which are based on a holistic system approach [12]. Given the limited adoption of OA’s key principles, we refer to organic by default when using the term OA in the context of Bhutan.

Due to the absence of agrochemical inputs in OA, access to nutrients for plant growth is limited and the occurrence of pests and diseases is more difficult to manage, resulting in lower yields than in conventional agriculture (CA). This is well documented for developed countries [2], but there is very limited research on OA’s relative productivity in developing countries generally, and in Bhutan specifically. Our study contributes to this research gap by estimating organic-to-conventional yield ratios of 16 annual and perennial crops using on-farm data from a nationally representative survey covering about 6,200 Bhutanese farmers.

The main contribution of this study is to assess the economy-wide impacts of Bhutan’s proclaimed 100% organic policy. Given agriculture’s critical role for the country’s economy, employing about 50% of the labour force in 2012 [13], significant effects of such an unprecedented national conversion policy on household welfare and food self-sufficiency are to be expected. Furthermore, the detailed representation of the agricultural sector in our model allows to investigate how the conversion affects agricultural output, land use, and the availability of crop nutrients.

## Policy background

The general concern for the environment is deeply anchored in Bhutan’s policies, for instance, by the constitutional mandate to maintain 60% forestry cover at all times [14] or the pledge to stay a carbon neutral country [15]. Part of the 100% organic policy rationale is to promote Bhutan as an organic brand, which shall help to commercialize smallholder agriculture, alleviate poverty, and add value to the tourism sector [16]. In order to become 100% organic, Bhutan’s government intends to “phase out [the] use of harmful chemical fertilizers and pesticides” [15], which effectively resembles a ban on agrochemicals. The use of mineral fertilizers compliant with IFOAM regulations (rock-phosphate and mineral potassium not enriched by chemical processes), do not play a role in Bhutan. Further, there is no domestic agrochemical production and the import of agrochemicals is a government controlled monopoly accounting for about 0.2% of total imports (in value terms) [17, 18]. Hence, a ban on importing or producing agrochemicals is relatively easy to implement and would only affect cropping systems directly, as animal husbandry in Bhutan follows traditional practices with negligible use of non-compliant inputs, such as artificial growth hormones.

Since the first announcement of the 100% OA objective in 2008 [11], the consumption of synthetic fertilizers used in Bhutan remained stable, while the use of pesticides even increased with an average annual growth rate of 11.8%, which is attributed to labour shortages [19, 20] (see S1 Fig). In 2012, 13,943 hectares were cultivated under CA, i.e., 18.6% of total cultivated land [10]. Of the remaining 61,194 hectares only about 545 hectares of crop land (less than 1% of total arable land) were certified organic [21] leaving the largest share of cultivated land to organic by default, i.e., no organic certification and no usage of agrochemicals, but also no use of improved organic practices. However, the Royal Government of Bhutan (RGoB) does not necessarily tie achieving 100% OA to governmental certification, except for exports, for which an internationally recognized certification is compulsory [19].

The implementation of the 100% organic policy in Bhutan is very different from policy measures addressing organic farming, e.g., within the Common Agricultural Policy of the European Union (EU). In the EU, conversion to certified OA is based on incentives like subsidies compensating for yield reductions and increase of labour costs while rewarding environmental and societal benefits of OA. Clear regulations were developed in close relation to the IFOAM norms [9] that define organic agriculture and the practices applied (e.g., EC No 834/2007 [22]). A conversion to OA in such a context is usually a conscious decision made by farmers, who are motivated by reasons of economic viability and their ethical values and often these actions are embedded in grassroots-movements [23, 24]. However, expert interviews with government representatives, researchers, and farmers conducted between 2014 and 2016 showed that Bhutan’s 100% organic policy is not the result of a bottom-up process involving farmers, but rather a top-down policy. An early report even noted: “farmers lack awareness on organic farming. They are even confused: why organic now when they have just learnt to practice conventional farming” [16].

The top-down nature of Bhutan’s 100% OA policy merits special emphasis. It is largely based on the phasing out of chemical inputs, but does not properly integrate measures that lead to the adoption of improved organic farming practices (e.g., composting, improved manure management, use of nitrogen (N)-fixing plants, management of biodiversity for bio control and others) as defined in the principles of IFOAM [9]. CA farmers in Bhutan would thus predominantly convert to organic by default and not by IFOAM principles. The relevant stakeholders do not seem to sufficiently discuss and account for this difference.

Bhutan, unlike many South Asian countries, has achieved a relatively high degree of food security as reflected by most nutrition indicators and in 2012, only 2.8% of the population were reported to live under the food poverty line of 2,124 kcal [25]. Yet, stunted growth in children younger than five years old and anaemia, which affects women and children, remain serious challenges [26]. Besides affecting food security, the OA policy could conflict with the objective of increasing self-sufficiency in cereals–a key priority of Bhutan’s agricultural policy. Through investments in mechanization and irrigation schemes, domestic paddy production shall be increased by 26% to achieve a rice-self-sufficiency rate of 67% until 2018 [8]. Considering the geopolitical tensions surrounding Bhutan, increasing food self-sufficiency is a valid national priority and this study will assess how this policy objective is affected by a 100% organic conversion.

## Materials and methods

We simulated the 100% organic policy in Bhutan as a phase-out of agrochemical-use, according to which conventional farmers adopt the current farming practices of their organic by default counterparts without introducing any technological advancement. This simulation only concerned the cropping sector as the livestock sector does not use any inputs that are non-compliant with OA. We followed a two-step approach. In step one, we utilized data from a nationally representative farm survey to test for differences in productivity of OA and CA within each of Bhutan’s three agroecological zones (AEZs). In step two, we employed a single country Computable General Equilibrium (CGE) model to simulate the economy-wide impacts of the 100% organic policy. CGE models are a common method used to assess the effects of policies and economic shocks, particularly in the field of agriculture and food policy, as they comprehensively depict the interdependencies in the entire economy. Both steps are linked by updating the model’s database, a 2012 Social Accounting Matrix (SAM) of Bhutan, using the results of the yield difference estimation.

### Data and estimation procedure of OA-CA yield differences

The analysis of yield differences is based on the nationally representative Agricultural Sample Survey (ASS) 2012 containing crop output data of about 6,200 farmers [10]. This dataset is the only recent survey that includes questions on agrochemical use in crops on a farm level. The dataset was obtained from the Ministry of Agriculture and Forests (MoAF) in Bhutan and was de-identified prior to access. Crops were classified as conventionally produced if farmers used chemical fertilizers, pesticides, or both on the crop area. The dataset was combined with altitude data on the sub-district (*gewog*) level to generate variables identifying the following three main AEZs. AEZ1 is the humid, sub-tropical zone at altitudes below 1,200 meters above sea level (masl). AEZ2 is the dry-subtropical AEZ in altitudes between 1,200 and 1,800 masl. AEZ3 is the temperate zone in altitudes above 1,800 masl.

Yield data, calculated by dividing production in kilograms by area in hectares, was cleaned by excluding the 1% and 99% percentile outliers within each crop and AEZ. Yield differences were calculated as an organic-to-conventional ratio per crop and AEZ level if at least 25 observations were available per cultivation system (OA or CA), otherwise the crop was excluded from the analysis. Observations of crops cultivated without agrochemical use (i.e., organically-grown crops) by farmers who utilized agrochemicals in other crops were also excluded in order to avoid any possible effect of nutrients from synthetic fertilizers via crop rotations and to avoid any effects of pesticide application or pesticide drift. Fieller confidence-intervals were calculated for the yield ratios and significance levels were tested conducting the non-parametric Wilcoxon rank-sum test on absolute yield levels. A non-parametric procedure was applied as the Shapiro-Wilk W test revealed that the majority of crop yields neither follows a normal nor a lognormal distribution.

The results of the yield gap analysis were utilized to update the model database. In case the yield difference was found to be significant (p-value < 5%), the cultivation system specific mean yields were used to compute total quantity produced per crop based on the distribution of land in the database among cultivation systems. If yield differences were not significant, the mean of all observations per crop across both cultivation systems was used.

### Economy-wide model framework

#### Model database

The 2012 SAM for Bhutan determines the structure of economic institutions and agents and is documented by Feuerbacher et al. [27]. The following two modifications were implemented:

Firstly, data from the ASS 2012 and the Bhutan Living Standard Survey 2012 [13] were used to disaggregate farm activities, factors, and households according to the three AEZs. Crop-producing activities were further disaggregated according to cultivation systems (i.e., OA or CA), which results in cultivation system and AEZ specific input-output structures. Secondly, eight field operation activities (e.g., manual weeding with or without pesticide use) were incorporated using available crop budget data. Each produces a corresponding field operation commodity that enters the crop producing activity as a production input. The field operation “Mechanical land preparation”, for instance, comprises ploughing and puddling of land using a powertiller requiring fuel, agricultural labour (*family and hired farm labour*), and agricultural capital (*powertiller*). The incorporation of field operations is a novel feature in CGE modelling. It allows simulations to explicitly model the trade-off between technological choices and directly links the labour requirement to each operation, rather than aggregating it.

Table 1 provides an overview of the SAM accounts. Farm activities comprise crop, livestock, post-harvest and community forestry activities and require farm production factors. Farm labour is either family labour (i.e., from the same household) or hired. We do not explicitly depict labour exchange groups, which is a common practice in Bhutanese agriculture, because the amount of labour supplied is typically close or equal to the labour received in return. There are two livestock accounts. Since other livestock than cattle (predominantly poultry, pigs, sheep, and goats) comprise only 5.2% of total livestock units in Bhutan [27], they are grouped together in the other animals account. In addition to farm labour and livestock there are also four agricultural land types, powertillers and other machinery. Income from these farm production factors accrues to either the farmer or landless households. We did not disaggregate by farm-size, which is rather homogenous in Bhutan, with more than 95% of farmers cultivating less than four hectares of land and a median landholding of about one hectare [10]. Non-agricultural households were differentiated by factor ownership and residence in either rural or urban areas. Agricultural households, activities and production factors were disaggregated by AEZ and in case of crop production and land also by cultivation system. In accordance with Bhutanese law, irrigated-land is only utilized by rice cultivation. Some activities have by-products such as crop residues, fodder, manure and draught animal services, which are used as inputs by other farm activities. Just as field operations, they are assumed to be non-tradable within Bhutan and thus were segmented according to AEZs. A table listing all SAM accounts in detail is provided in the supporting information (S1 Table).

**Table 1:** Disaggregation of 2012 Bhutan SAM.

Category	Item	
Activities	Agriculture:	Crops^1^ (7), Livestock^2^ (2), Field operations^1^ (9), Community forestry^1^, Commercial forestry, Post-harvest processing^1^ (5)
	Industry:	Extractive industries, Food processing, Textile, Other manufacturing, Public utilities
	Services:	Construction, Trade, Transportation, Hotel and restaurants, Government services, Other services
Factors	Labour:	Skilled, Unskilled, Family farm^2^, Hired farm^2^
	Land:	Rainfed land^1^, Irrigated land^1^, Orchards^1^, Pasture land^2^
	Capital:	Private capital, Public capital, Informal capital, Powertiller^2^, Other machinery, Cattle^2^, Other animals^2^
Margins	Trade, Transportation	
Institutions	Agricultural households:	Farmer, Landless
	Non-agricultural households:	Urban skilled, Urban unskilled, Rural skilled, Rural unskilled, Capital dependent, Transfer dependent
	Private enterprises, Public enterprises, Government, Rest of the world (Row)	
Taxes	Import tax, Excise tax, Sales tax, Income tax, Production tax	
Capital	Investments, Stock changes	

Note: Number in parenthesis describes the quantity of items, e.g. there are seven crop sectors.

^1^ Disaggregated by the three agroecological zones and cultivation system

^2^ Disaggregated by the three agroecological zones

#### Behavioural relationships in the model

A CGE model captures the entire circular flow within an economy, as illustrated by the following example. A farmer household located in the low-altitude zone (AEZ1) allocates land and labour to an AEZ-specific cropping activity from which it receives a factor rent in return which finances its expenditure for consumption, taxes, transfers, and savings. The cropping activity receives income from the sale of its output, which is again needed to finance the use of intermediate inputs and factors. Hence, each agent or institution expenditure represents income somewhere else within the circular economy.

According to our model setup, each crop can be produced by two cultivation systems (OA vs. CA) across the three AEZs and a Constant Elasticity of Substitution (CES) demand function is utilized to aggregate total supply across all activities producing the same crop. We assumed no price premiums for OA goods. Organic and conventional output are instead perfect substitutes produced with different technology and cost structures. There has been hardly any research assessing the Bhutanese consumers’ willingness to pay a price premium for OA produce. For the case of organic rice, no significant price premium was found to be paid by Bhutanese consumers [28]. Due to the lack of a domestic market for officially-certified organic products, this finding can be generalized for all crops that are produced under OA.

Agricultural exports could potentially benefit from price premiums. Yet, except for a few minor export items, such as lemongrass oil, the vast majority of exports are not sold as organic products, even though cardamom and citrus fruits (two of the three most important agricultural export items) are produced without agrochemical use. Moreover, the purchasing power of the current main export markets, India and Bangladesh, which absorb 90% of total agricultural export value [18], is rather low and hence we do not assume a price premium for export goods. While the domestic market for organic products is not established yet, there is a strong awareness of Bhutanese consumers to distinguish between domestic and imported produce. This becomes apparent when visiting the country’s largest market, the Centenary Farmers Market in the capital city Thimphu, where Indian imports are sold at the ground floor and only Bhutanese products are allowed to be sold on the first floor. Using the Armington assumption [29] that makes imports imperfect substitutes for domestic products, we were able to reflect this pattern of consumer preference in our model.

The CGE model adjusted for this study is the single country, comparative-static STAGE2 model which is comprehensively described in McDonald and Thierfelder [30]. The institutions and agents in the model are production activities, commodity markets, households, incorporated enterprises, the government, and the capital market. All markets are assumed to be perfectly competitive where consumers maximize utility and producers’ profits and prices adjust to clear markets. Household consumption follows the Linear Expenditure System according to which households maximize their utility from consumption of which a fixed part represents vital subsistence consumption.A large share of what Bhutanese farmers consume is their own produce. This is not explicitly reflected in the model. Instead, farmers purchase their own output through the commodity market.

Activities describe the domestic production of commodities, which combine fixed shares of intermediate inputs (i.e., inputs required for the production process) and production factors (i.e., labour, capital and land) and transform them to outputs. In contrast to intermediate inputs, factor inputs can be substituted with each other to differing degrees (implemented as multi-level CES functions). The substitution possibilities between factors is determined by a production structure, which was extended to incorporate field operations. This novel approach allows to explicitly model technological trade-offs within the agricultural sector, such as land preparation using either manual labour, draught power or powertillers. It also includes a nest governing the substitution of cultivated land and fertilizer input, for which we use a low substitution elasticity of 0.4 as proposed by [31] in the context of developing countries. A detailed documentation of the model’s production structure and parameters is provided in the supporting information (S1 Text).

#### Model closures

Since Bhutan is a small country, the model assumes fixed world market prices. The external exchange rate is fixed. This reflects Bhutan’s current currency regime of a one-to-one peg of the Bhutanese Ngultrum with the Indian Rupee. India is by far Bhutan’s most important trade partner, accounting for more than 78% of imports and 94% of Bhutan’s exports in 2012 [18]. The consumer price index (CPI) is set as the model’s numeraire. The model is investment-driven (investment is fixed as a share of final demand) with equi-proportionately varying saving rates for households and enterprises. Government consumption and savings are fixed in quantity terms, and the government account is balanced by relative changes in the income tax rate.

Capital supply is constant and assumed to be immobile and activity specific as we consider a short-term adjustment horizon. Skilled and unskilled labour are perfectly mobile across activities. Agricultural labour, both family and hired farm labour, are segmented according to AEZs, and thus only mobile within the activities of the same AEZ. The three land-types (irrigated land, rainfed land, and orchard) are set immobile across AEZs and cultivation systems, establishing the entry point of the shock of converting to organic agriculture. Unlike the other factors, the model closure for land accounts for unemployment, as a significant share of total arable land was left fallow in the base period. The land supply regime is explained within the next section.

#### Modelling approach

To achieve Bhutan’s 100% organic policy, a likely real-world policy instrument would be a ban on the use of agrochemicals. In a CGE model, such a policy is typically mimicked using a prohibitive tax (e.g., import or sales tax) which increases prices such that economic agents are incentivized to entirely substitute the use of the taxed good [32]. This approach has the disadvantage that a very high distortion of input prices would be needed in order to achieve a scenario close to 100% conversion. Therefore, a novel approach is applied in this study, modelling the phase-out of CA as a conversion of conventional to organic land within each AEZ and land type.

Eq 1 describes the converted quantity of conventional to organic land *CVT*0_*on*_, which is the base supply of conventional land *FS*0_*cn*_ multiplied by the share of conversion *shrcvs* and the diagonal matrix *mapland*_*cn*,*on*_ to map organic *(on)* and conventional *(cn)* land-types according to AEZ and land type. Due to technical reasons, *shrcvs* is limited to 99.99% to simulate Bhutan’s 100% organic policy.

$${CVT0}_{on}={FS0}_{cn}*shrcvs*{mapland}_{cn,on}$$ (1)

Organic activities may not absorb all converted land and the portion of unutilized converted land is captured by the variable *CVI*_*on*_. Eq 2 formally describes the binary mechanism that determines the factor price *WF*_*on*_ for organic land types *(on)*.

$$\frac{{WF}_{on}}{CPI}=\left\{ \begin{array}{c} \frac{{WF0}_{on}}{CPI},if\;{FW}_{on}\geq {FW0}_{on}\;or\;{CVI}_{on}>0\\ {\left(\frac{{FS0}_{on}+{(FW0}_{on}-{FW}_{on})}{{shfs}_{on}}\right)}^{\frac{1}{{elafs}_{on}}},if\;{FLW}_{on}<{FW0}_{on}\;and\;{CVI}_{on}=0 \end{array}\right.$$ (2)

where *FS*0_*on*_ is the organic land supply in the base period, *FW*0_*on*_ is the base quantity of fallow land, *FW*_*on*_ is a variable for fallow land, *shfs*_*on*_ is a calibrated shift parameter, and *elafs*_*on*_ is the supply elasticity of land which is set to 0.1. There are no specific land supply estimates for Bhutan, but the selected elasticity fits well within the range of land supply elasticities applied throughout the literature [33]. The land price, deflated by the CPI, is assumed constant (*WF*0_*on*_) if the total supply of land within each AEZ and land-type is equal to or smaller than base supply (i.e., supply at perfect elasticity). However, land prices increase according to an upward sloping land supply curve once fallow land from the base period is put under cultivation, which then represents a land expansion within a specific AEZ and land-type.

## Results

As previously mentioned, we applied a two-stage approach. In this section, firstly the results of the yield estimation are provided, which establish the entry point for the model analysis. Secondly, we present the results of the economy-wide model in detail.

### Yield differences between OA and CA

**Fig 1:**
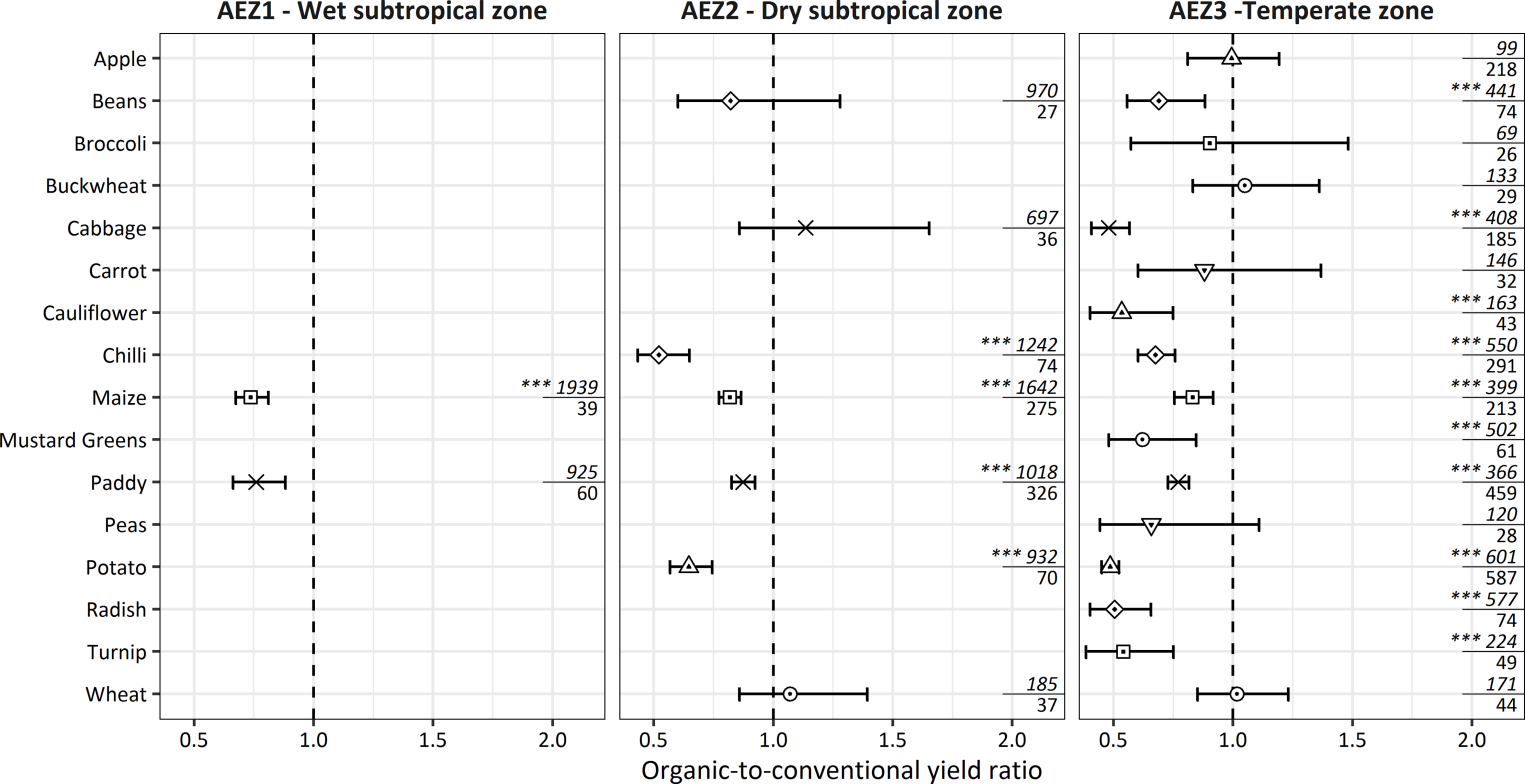
Organic-to-conventional yield ratios for 16 crops across agroecological-zones in Bhutan based on data from [10]. The panel on the right-hand side shows the significance level of the Wilcoxon rank-sum test (*** P < 0.001) and the fraction shows the number of organic (numerator) and conventional (denominator) observations.

The yield difference estimation shows that conventional yields are mostly higher than organic yields (i.e., yield ratios below one). Differences are statistically significant for 15 out of 25 yield comparisons all at the 0.1% significance level (Fig 1). The prevalence of CA is highest in AEZ3, with 46.5% of cultivated area under CA. These shares are much lower in AEZ2 (12.1%) and AEZ1 (1.4%), which explains the lower number of yield comparisons in these AEZs.

The estimated ratios for paddy, which are of particular importance given Bhutan’s focus on rice self-sufficiency, are only statistically significant within AEZ2 and AEZ3, where 70.2% of paddy is produced. For paddy in AEZ1, the Wilcoxon rank-sum test does not yield a significant difference although the confidence interval is below the unitary ratio. Table 2 provides an overview of shares of conventional production across crops and regions after adjusting for significance in yield differences. Except for the major crops (i.e., paddy, maize, and potato), crops are aggregated in the SAM, which is why yield differences of minor crops (e.g., cabbage) are not directly traceable in the model.

**Table 2:** Percentage shares of crop output value in model database.

Crop / Region	Share of conventional agriculture in production per crop and within region				Share of crop in total crop production value
	AEZ1	AEZ2	AEZ3	National	
	(%)	(%)	(%)	(%)	(%)
Paddy	3.3	29.2	61.1	30.4	22.0
Maize	1.3	12.3	63.8	22.0	12.8
Other Cereals*	0.0	3.3	8.3	4.3	7.2
Vegetables	0.0	8.9	29.6	18.6	15.1
Potato	0.0	16.5	88.5	77.1	12.9
Spices	0.0	0.0	0.0	0.0	9.1
Fruits	2.6	1.9	63.1	15.0	20.9
**Total crops**	**1.7**	**13.0**	**57.1**	**25.7**	**100.0**

Columns 2–5 refer to the share of conventional crop production per crop type and region. Column 6 refers to each crops’ share in total crop output value on the national level independent of production system (CA and OA). AEZ1-3 are the three main agroecological zones in the low-, mid-, and high-altitude ranges.

*Includes legumes and oilseeds

The relevance of crop yield differences for the economy-wide simulations in step two depends on the share produced by CA and a crop’s relative weight within the agricultural sector. Table 2 shows that according to both criteria, paddy, maize, and potato have the highest shares produced by CA and are also among the most important crops accounting for 48% of total crop output. In AEZ2 and AEZ3, these crops also have large and significant yield gaps (Fig 1). In contrast, the conventional production of other crops (e.g., vegetables) is of very small magnitude and for spices it does not even exist.

### Model results: Economy-wide changes of the 100% OA policy

#### Macro-level changes

The large-scale exogenous conversion of conventional land constitutes a negative factor endowment shock due to generally lower yields within OA. In the base, all conventional land (13,943 hectares) accounted for 18.6% of total cultivated land, but comprised a disproportionately higher share of total land returns (24.3%).

**Fig 2:**
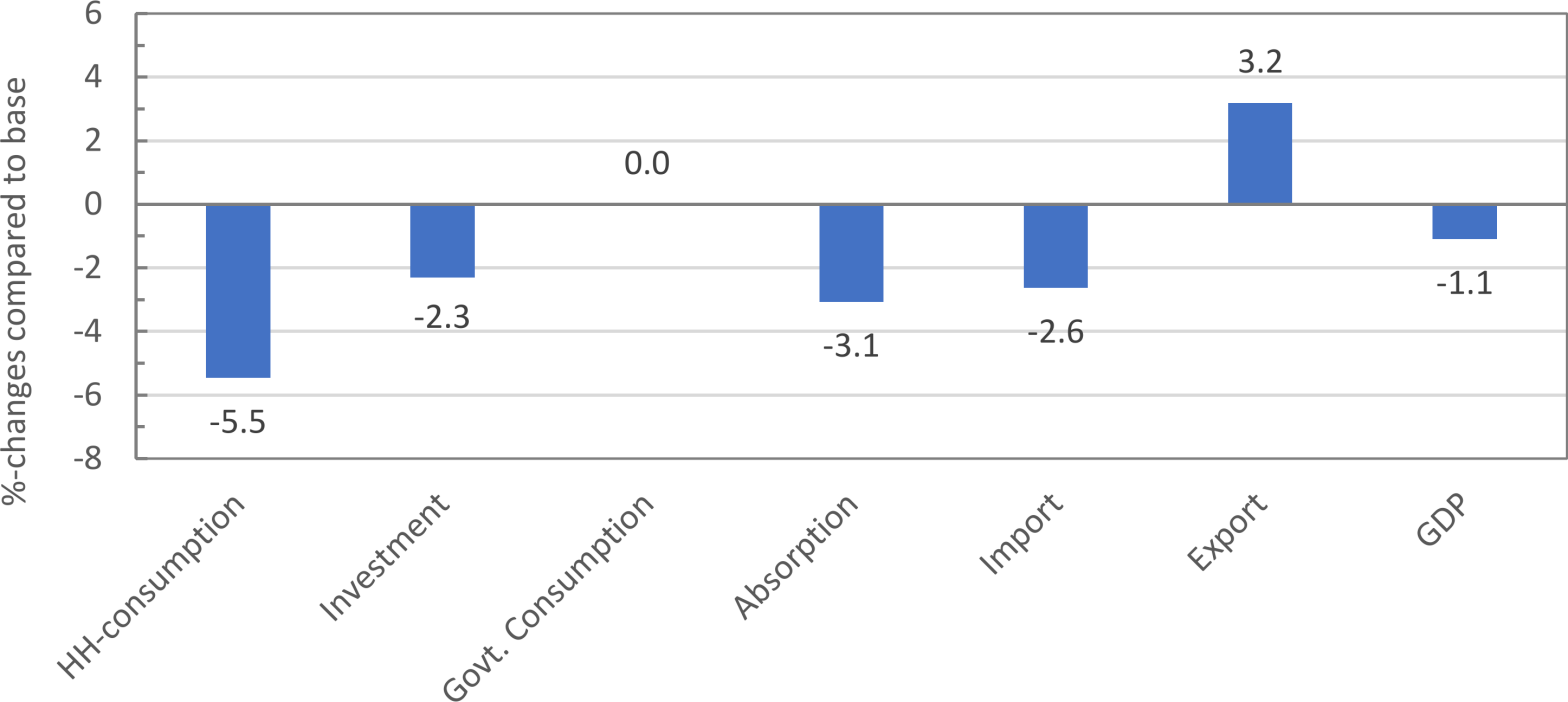
Percentage changes of macro indicators in real terms after simulating a 100% organic policy.

Consequently, the 100% organic policy results in decreasing overall economic activity (Fig 2). Households experience a loss in purchasing power as factor income declines, food prices surge and household consumption shrinks accordingly (-5.5%). Investments as a fixed share of aggregate demand decrease (-2.3%). Fixing the government demand in quantity terms results in constant government expenditure in real terms. In line with falling household demand total imports also decrease, however, to a lower extent (-2.6%). Exports increase (3.2%) as decreasing non-agricultural factor prices raise the competitiveness of export industries. Net savings abroad increase (10.8%) as a result of a lower current account deficit. Overall, the model results suggest that Bhutan’s economy would experience a drop in real GDP of -1.1% and a drop in total domestic absorption by -3.1%.

#### Changes in factor markets

After the conversion, total cultivated land increases slightly by 0.8%. The OA policy affects land-types very differently. Total irrigated land and orchards decline (-6.7% and -2.7%), while the cultivation of total rainfed land increases (4.0%). An overview of the converted land per land type and AEZ is provided in Table 3. The largest share of conversion occurs in the AEZ3, while the shock affects only a small share of cultivated land in AEZ2 and particularly AEZ1, where most agriculture is already organic by default. Previous fallow land is cultivated in addition to reallocated conventional land in those cases where changes in cultivated organic land after the conversion (reported in column five of Table 3) are larger than the convertible conventional land (column four). This holds, for example, for irrigated land in AEZ1, for orchard land in AEZ1 and AEZ2, and for rainfed land across all AEZs. Cultivated organic irrigated land only increases in AEZ1 and not all previous conventional irrigated-land is cultivated in AEZ2 and AEZ3.

**Table 3:** Reallocation of conventional land and change in cultivated organic land (based on the reference year 2012).

AEZ	Land type	Organic land in base	Converted conventional land	Chg. in cultivated organic land	Chg. in total cultivated land
		(ha)	(ha)	(ha)	(%)
AEZ1	Irrigated land	5,446	186	260	1.3
	Rainfed land	14,779	92	831	5.0
	Orchard	5,314	86	118	0.6
	*Sub-Total*	*25*,*539*	*364*	*1*,*210*	*3*.*3*
AEZ2	Irrigated land	5,045	1,831	1,435	-5.8
	Rainfed land	16,599	1,408	2,335	5.1
	Orchard	2,181	36	48	0.5
	*Sub-Total*	*23*,*824*	*3*,*275*	*3*,*818*	*2*.*0*
AEZ3	Irrigated land	1,869	2,301	1,506	-19.1
	Rainfed land	9,141	7,512	7,835	1.9
	Orchard	820	533	247	-21.1
	*Sub-Total*	*11*,*830*	*10*,*347*	*9*,*588*	*-3*.*4*
National	Irrigated land	12,360	4,318	3,202	-6.7
	Rainfed land	40,519	9,013	11,001	4.0
	Orchard	8,315	655	413	-2.7
	**Total**	**61,194**	**13,986**	**14,616**	**0.8**

AEZ1-3 are the three main agroecological zones in the low-, mid-, and high-altitude ranges. Column three reports cultivated organic land in the base and column four the total conventional land available for conversion. Column five describes the net absolute change in organic land after converting all or a share of conventional land. If all conventional land is converted, then previous fallow land can be cultivated. In this case, a positive change in total cultivated land is reported in column six.

The lower yields of OA paddy reduce the use of irrigated land, since this land can only be used for the cultivation of paddy, but not for other crops as defined by the current legal setting in Bhutan. This is particularly relevant in AEZ2 and AEZ3, where organic yields are significantly lower than yields in CA. In contrast to irrigated land, farmers can cultivate a wide range of crops on rainfed land and in orchards, which allows them to specialize in those crops which have the highest productivity under the given conditions. Thus, the demand for rainfed land increases in all AEZ. The reduction in the cultivation of total orchard land should be interpreted as a drop in utilization (i.e., less orchards are managed and harvested) and is driven by the strong drop in AEZ3, where apple cultivation depends on agrochemical use.

Table 4 shows the changes in factor prices. Due to the inelastic land supply curve, land prices increase strongly once all fallow land is cultivated. This is particularly the case for rainfed land across all AEZs. Despite higher labour intensity within cropping activities, overall agricultural wages decrease due to the lower productivity of OA. Aggregate labour absorbed by cropping activities in AEZ1 and AEZ2 increases by 4.8% and 5.8%, respectively, while in AEZ3 it drops by -4.7%, which results in an aggregate increase of 2.3%. The additional agricultural labour demanded by cropping activities is released by post-harvest and off-farm activities. These either require less labour because of falling output prices reflecting lower demand for their final outputs (e.g., forestry and weaving of textiles) or because of decreased availability of necessary inputs (e.g., milling of cereals). The overall reduction in household demand results in a decrease of non-agricultural output, which has negative consequences for non-agricultural labour and capital.

**Table 4:** Percentage changes in factor prices after simulating a 100% organic policy.

Production factors		AEZ1	AEZ2	AEZ3	National
		(%)	(%)	(%)	(%)
Agricultural production factors	Irrigated land	13.4	0.0	0.0	4.9
	Rainfed land	61.0	70.3	39.7	57.4
	Orchard	5.1	4.6	0.0	4.4
	Pasture land	4.6	4.9	2.8	4.0
	Family farm labour	0.6	0.6	-2.9	-0.6
	Hired farm labour	-0.2	-1.0	-2.8	-1.4
	Powertiller	9.7	8.0	-9.9	-0.1
	Cattle	4.6	4.9	2.8	4.0
	Other animals	-1.5	-1.5	-0.3	-1.3
	Other machinery	-	-	-	-8.1
Non-agricultural production factors	Skilled labour	-	-	-	-8.1
	Unskilled labour	-	-	-	-5.4
	Private capital (factor income)	-	-	-	-4.6
	Public capital (factor income)	-	-	-	-0.3

AEZ1-3 are the three main agroecological zones in the low-, mid-, and high-altitude ranges.

#### Changes in agricultural output

**Fig 3:**
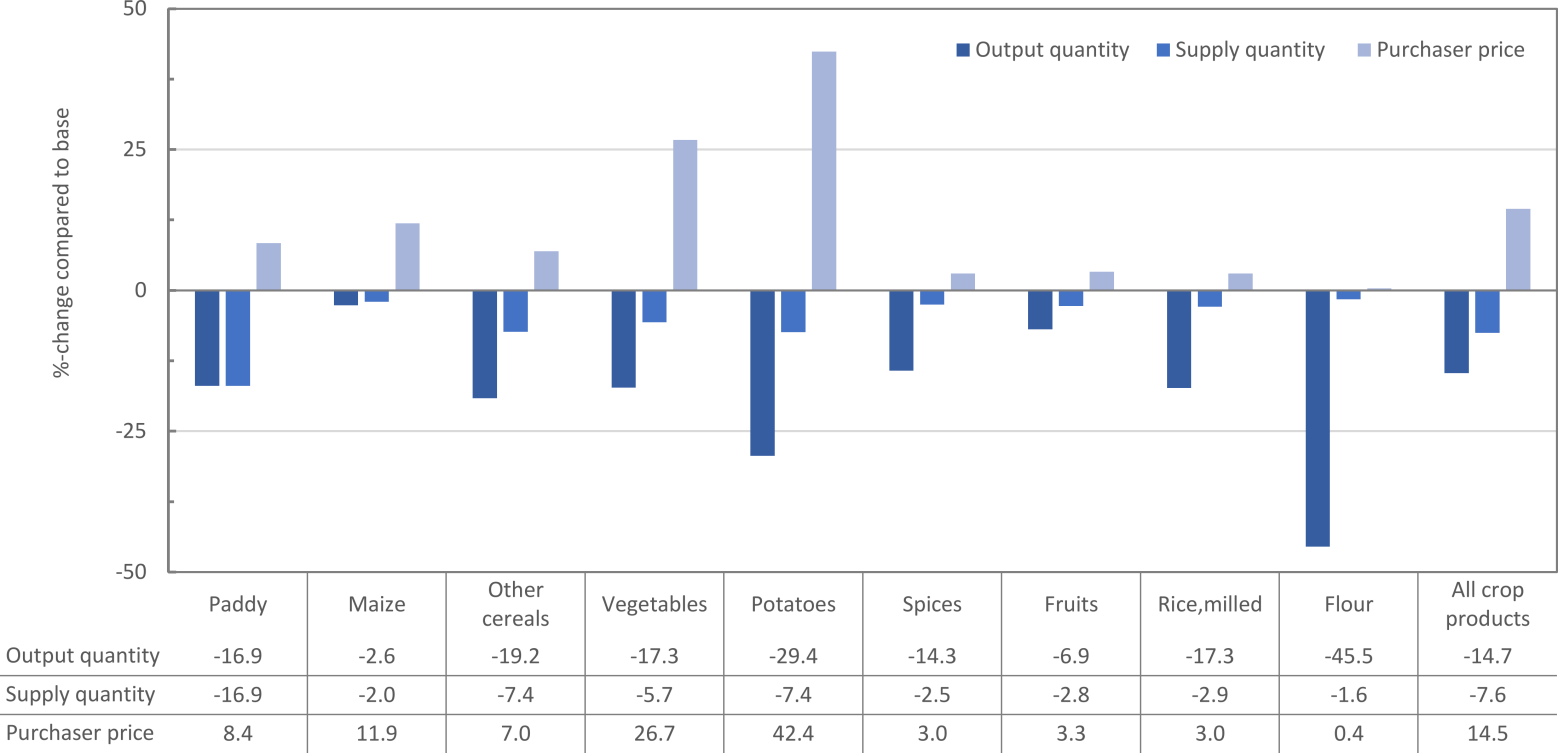
Percentage changes in crop output, supply, and purchaser prices after simulating a 100% organic policy.

Aggregate crop output declines by -14.7% in quantity terms resulting in a strong increase of crop prices (Fig 3). The reduced output in paddy and potato explains the largest part (52.4%) of the decline in crop output. Strong reductions in output are also observable for other cereals, spices, and fruits, however; these crops have lower shares in total production. Only 3.1% of domestic maize demand is imported and household demand for maize was estimated to be extremely inelastic reflected by an income elasticity just above zero [34]. Due to these specific trade linkages and consumer preferences maize production drops only modestly (-2.6%).

In many other cases, reductions in output are absorbed by trade. The mechanism by which output reduction is absorbed by either or both increased imports or decreased exports is dependent on the initial trade linkage measured as a percentage of base supply. In quantity terms, crop imports increase by 20.7% largely caused by higher imports of potatoes and vegetables. Considering all food imports, the strong increase of milled rice imports (16.1%) represents in absolute terms the largest increase of any food item. Total crop exports experience a significant drop (-29.8%), of which 56.8% is due to the reduction in potato exports. As a result of both declining exports and increased imports of traded crops, supply decreases at much lower rates than production (Fig 3).

Purchaser prices and output of edible livestock products decrease because of lower household demand (See S2 Fig for more details), and, in case of output because of higher demand for complementary non-edible livestock output (i.e., manure and draught power). Since the stock of animals is fixed exogenously, the supply of manure is inelastic. Consequently, following the conversion, manure prices increase strongly across AEZ (13.9%, 19.0% and 21.4% in AEZ1, AEZ2, and AEZ3, respectively). An increase in manure supply depends on changes in livestock management systems that result in an increasing recovery rate of manure droppings (e.g., by changes in animal husbandry from extensive pasture systems to indoor stable systems). This is reflected in the model, by allowing a low substitution elasticity between cattle and labour in the production structure and by using a CET specification to govern the output composition, which allows for higher output shares of manure and draught power while the output of edible livestock products decreases. Livestock product imports decrease as purchaser prices drop and total domestic production including edible and non-edible products slightly increases.

The lower drop in livestock production dampens the reduction of crop output, resulting in a decline of aggregate agricultural production of -10.6%. On the aggregate level, total food production (which includes processed food and excludes non-edible output) falls by -9.3%. Food supply is only reduced by -3.6% thanks to the increase in food imports.

#### Changes in agricultural inputs

The phase-out of agrochemicals results in a strongly increased demand for organic plant protection and organic fertilization (Table 5). The effects are most pronounced for AEZ3 where the share of converted conventional land is highest. Due to a slight increase of total land use in AEZ1 and AEZ2, total demand for field operations increases. In AEZ3, where total cultivated land decreases, demand declines for mechanical land preparation, manual land preparation, and other operations.

**Table 5:** Percentage changes of field operation supply.

Field operations / Agroecological zones	AEZ1	AEZ2	AEZ3
	(%)	(%)	(%)
Mechanical land preparation	0.2	0.2	-0.2
Draught animal land preparation	5.5	4.3	3.1
Manual land preparation	48.9	66.9	-1.2
Organic fertilization	6.1	5.7	8.8
Chemical fertilization	-100.0	-100.0	-100.0
Other operations	4.3	6.1	-7.3
Organic plant protection	9.9	24.8	95.3
Chemical plant protection	-100.0	-100.0	-100.0

AEZ1-3 are the three main agroecological zones in the low-, mid-, and high-altitude ranges.

The intensity of fertilizer use decreases. While there is a slight land expansion, the aggregate supply of the nutrients N, phosphorus (P), and potassium (K) declines by -6.4% from 105.6 to 98.8 kg ha^-1^, due to the abolishment of most synthetic and mineral fertilizers. In the base situation, synthetic and mineral fertilizers contribute with 26.9%, 6.7%, and 2.6% to the total supply of N, P, and K, respectively. This can only be partially replaced by an increased use of manure, which has a nutrient content of 1.6% N, 0.8% P, and 2.9% K (on dry matter basis) on average in Bhutan [35]. Given that N comprises 81.4% of the NPK content in previously used synthetic fertilizers, availability of N drops significantly (-22.4%) and the average N application rate falls from 38.4 to 29.8 kg ha^-1^. The absolute amount of P applied remains almost stable at 15.0 kg ha^-1^ (-0.1%) and the use of K increases slightly by 3.4% to 54.0 kg ha ^-1^. The NPK composition in the base of 36.3% N, 14.2% P, and 49.4% K changes to 30.2% N, 15.2% P, and 54.6% K.

#### Changes in food consumption and welfare

Following a 100% organic conversion policy, national food self-sufficiency declines by -5.1% from previously 84.1% to 79.0%. Self-sufficiency is computed by dividing the value of domestic production by the sum of the values of domestic production and imports minus exports using constant prices of the base period. Cereal self-sufficiency is reduced by an even higher rate (-6.3%) from 61.5% to 55.2%. The decline in cereal self-sufficiency is largely driven by the substantial drop in paddy output and the increase in rice imports. Agricultural households experience a decrease in food consumption by -2.3% on average with considerable regional differences. Farmers’ consumption in the low-altitude zone (AEZ1) remains constant, while farmers in the temperate zone (AEZ3) decrease consumption by -5.6% (Table 6). Non-agricultural households face higher reductions in income and thus on average reduce food consumption at a higher rate (-5.2%).

**Table 6:** Percentage changes of household income and food consumption for different income types and agroecological zones.

Household types	Household income	Food consumption
	(%)	(%)
*Non-agricultural households*	*-5*,*3*	*-5*.*2*
Urban skilled	-5.2	-5.0
Urban unskilled	-5.9	-5.3
Rural skilled	-5.1	-5.5
Rural unskilled	-5.7	-5.6
Other income	-4.6	-4.5
*Agricultural households*	*-1*.*9*	*-2*.*3*
AEZ1 farmer	2.5	0.0
AEZ2 farmer	1.0	-0.8
AEZ3 farmer	-7.3	-5.6
AEZ1 landless	-3.0	-3.9
AEZ2 landless	-2.9	-3.0
AEZ3 landless	-4.8	-4.4
**National**	**-4.3**	**-3.9**

AEZ1-3 are the three main agroecological zones in the low-, mid-, and high-altitude ranges.

**Fig 4:**
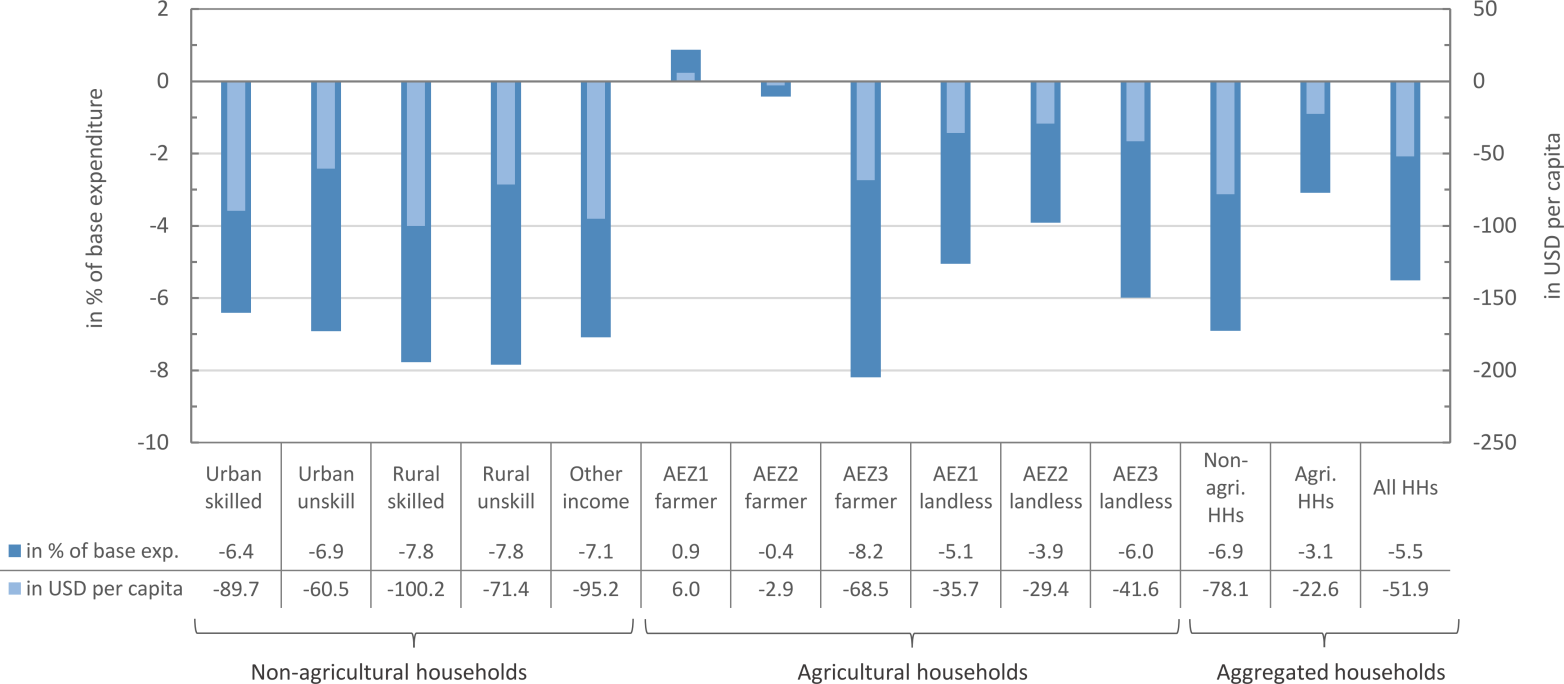
Welfare effects of Bhutan’s 100% organic conversion policy. Changes in welfare are measured as equivalent variation (EV) as share of base household expenditure on the left-hand side and in USD per capita on the right-hand side.

Welfare, measured either in USD per capita or the share of equivalent variation in base household expenditure, decreases for all households, except for agricultural households in AEZ1 (Fig 4). The average Bhutanese bears a welfare loss of -51.95 USD or -5.51% in terms of base household expenditure. Agricultural households both experience lower welfare losses in relative and absolute terms compared to non-agricultural households. The strongest relative welfare-decline; however, is incurred by farmers in AEZ3, where most of the agricultural output is reduced causing lower factor income from labour and land (particularly irrigated land). In AEZ1 and AEZ2, landless households suffer from higher welfare losses than farmer households, because land rents increase while wages remain stagnant or even decline. Farmer households in AEZ1 even show a slight benefit, as they receive higher factor income from labour and land. In addition to these welfare changes at the household level, positive welfare effects result from the reduced current account deficit, which implies lower foreign debt and reduced interest payments in future periods. Using an alternative closure with a fixed current account balance, but flexible exchange rate, welfare of households on average drops by -2.1% of base income, while real GDP drops at a similar rate (-0.9%).

## Discussion

### Discussion of estimated yield ratios

Based on our data the estimated yield gap between OA and CA is the key input determining the model results. Our estimated simple average organic-to-conventional-ratio of 0.76 compares well with recently estimated global mean ratios that range between 0.75 and 0.81 [2, 5, 6]. Yield ratios in developing countries are particularly controversial due to data scarcity. Ponisio et al. [2] and Ponti et al. [6] do not find any statistically significant differences of yield ratios between developing and developed countries. In contrast, Seufert et al. [5] find a significantly lower ratio of 0.57 for developing countries compared to 0.80 for developed countries.

Our yield ratio estimates for paddy and most other major crops seem to be plausible when compared to literature. The paddy OA-CA yield ratios for AEZ2 (0.87) and AEZ3 (0.77) are at the lower end or respectively below the range of ratios (0.86 to 1.05) reported by Ponti et al. [6]. But for the main three field crops in Bhutan (paddy, maize and potato), our yield ratios estimates fall well within the range of ratios provided in the supplementary materials of Ponisio et al. [2, 36]. Arguably, yield ratios derived from literature can only serve as a limited point of reference to validate our results due to limited comparability. Yet, our results are also corroborated by field trial evidence showing that yields in the current low-input farming system in Bhutan would increase through (higher) application of agrochemicals [35, 37, 38].

Due to the lack of data, critics often highlight that yield ratios in developing countries are potentially biased, because in some cases low-input conventional systems are compared with optimized OA systems [3], while very low ratios are partially explained by atypically high conventional yields [39]. To date, there have been no field trials of experimental stations or controlled on-farm trials in Bhutan that studied the yield difference between OA and CA generally or specifically for different farming intensities. Field trials in Kenya [40, 41] analysing organic and conventional farming systems of different intensities (mainly N input and rotation design) find yield differences in OA and CA with OA < CA or OA = CA or OA>CA depending on fertiliser inputs, management practices, crop species, and location. We therefore assume that under Bhutanese conditions similar context dependent results are to be expected. The only study assessing organic and conventional yields in Bhutan analysed rice grain yield data from 120 farms evenly sampled across all three AEZs in 2012 and 2013 [28]. Using ANOVA models the authors could not find any statistically significant differences between organic and conventional yields. This finding clearly differs from our results, as we find organic paddy yields in AEZ2 and AEZ3 to be 13% and 23% lower. Applying the same procedure (ANOVA) on our 2012 dataset, we find the yield difference of paddy even in AEZ1 to be statistically significant at the 1% level.

The dataset used to estimate the yield gaps is the only representative farm survey conducted in Bhutan with crop specific questions on agrochemical use. However, it lacks further relevant information on variables that might influence yields, such as farmer’s education, asset ownership, intensity of input use as well as the slope of fields and the type of soils, which could have been used for regression analyses. To assess the robustness of our results with respect to any potential upward or downward biases of the yield estimates, we conducted a sensitivity analysis of the yield parameters.

### Discussion of model results

Only a few studies have previously assessed the impacts of a large-scale conversion. Most of these are rather motivated by stylized thought-experiments using model approaches without accounting for economy-wide effects [42, 43]. The only comparable study simulates a 100% conversion for the Danish agricultural sector employing a comparative-static, single-country CGE model [44]. Under similar conditions, i.e., assuming no improvement in organic yields after conversion and allowing imports of animal feed, the authors find agricultural production to decline by -20.4% compared to the simulated decline of -10.6% for Bhutan. While organic farming practices might be more advanced in developed countries, there is also a greater dependency on agrochemicals, which could explain the comparatively lower drop in agricultural production for the case of Bhutan. Moreover, unlike developed countries, Bhutan’s livestock sector is predominantly ruminant based [45] and relies only to a small degree on conventional inputs and concentrate feed [46] and thus is only indirectly impacted by a 100% conversion.

Looking at the economy-wide implications of a 100% OA conversion, the contraction in crop output leads to negative economic growth (-1.1% decline in real GDP or about 18.8 million USD). More than half of the reduction in crop output is due to the strong reduction in domestic production of paddy and potato, both of which are crops with large initial shares of conventional production and high yield gaps. Additionally, consumer preferences and trade linkages matter, as the example of the moderate decline in maize production shows. Trade also appears to buffer the drop in domestic production as reflected by the strong decline of the food self-sufficiency rate. In this context, the decline in paddy output appears to be most sensitive, which highlights the need to devote more resources to increase efficiency in organic paddy production, if the current degree of self-sufficiency is to be maintained. It is important to emphasize, that a 100% organic conversion on a country-level with the opportunity to import more conventionally produced food creates leakage of environmental externalities in the countries, which produce the additional imports.

The reduction in GDP and household welfare is considerable given that the agricultural sector comprises only about 10% of GDP in 2012. From a distributional point of view, the cost of the 100% conversion would largely be borne by non-agricultural households. Among agricultural households particularly farmers in the temperate zone (AEZ3) would suffer from declining welfare. The welfare effects depend on the degree to which households are willing to substitute domestic food with imported food, since the price level of the latter is not impacted. The chosen model setup aggregates imports and domestic goods according to a national substitution elasticity, without considering household specific preferences.

Referring to the four pillars of food security [47] our results show adverse impacts for food availability, access, and stability. Our model does not capture changes within the fourth pillar, food utilization (i.e., the metabolism of food by individuals) [48]. Food availability corresponds to the supply of food, which declines at the aggregate level by 3.6% and in case of crops by even 7.6%. Also, food access drops markedly as shown by the decrease in food consumption by households. Hence, the 100% OA policy is likely to partially reverse Bhutan’s past achievements in terms of food security and to jeopardize households close to or under the food poverty line of 2,124 Kcal. Our results show an increased dependency on food imports from India, which can have ambiguous effects on food stability. Stronger dependency on food imports might make the country less vulnerable against harvest failures within Bhutan, yet also increases the exposure to possible changes in India’s trade and foreign policy.

From an agronomic perspective the model results are in line with ex-ante expectations regarding the impacts on land use and nutrient availability. Overall, the land area under cultivation increases, but only due to increased cultivation of rainfed land, while cultivation of orchards and irrigated land declines. In case of irrigated land, this is not only due to declining competitiveness of paddy cultivation, but also due to the Bhutanese law prohibiting other crops to be grown on irrigated land, which is a policy to foster rice-self-sufficiency. If other crops were allowed to be cultivated on irrigated-land, then paddy cultivation would likely decline further as irrigated-land would increase in price. Yet, in reality, many farmers cultivate other crops besides paddy on irrigated-land. Therefore, we probably underestimate the effects of the 100% organic policy on rice self-sufficiency.

A key challenge of OA is the supply and availability of crop nutrients, particularly N. As the organic fertilizers (e.g., compost, animal manure) permitted in OA provide a lower nutrient density and a slower nutrient release compared to synthetic fertilizers, nutrient availability in OA often does not match plant needs [49]. Animal manure, predominantly from cattle, mixed with crop residues (i.e., farmyard manure) is the most important organic fertilizer in Bhutanese agriculture. Forests and grasslands account for about 22% and 53% of cattle’s feed requirement, respectively, allowing for a nutrient transfer to cropland [46, 50]. Yet, as cattle husbandry is often not stable based, animals either graze freely or are tethered on site. Thus, there is the potential for increased targeted nutrient transfer to crop land through improved animal husbandry systems, which has to comply with OA standards for animal welfare. After the simulated conversion, the availability of P and K remain stable or even increase. Animal manure cannot replace the previous share of N from synthetic fertilizers (27%), resulting in a decline in N availability (-22.4%).

Our analysis assumes the initial headcount of livestock to be fixed, which limits the potential of additional manure production. In organic farming systems, the only source of N inputs that can replace synthetic N fertilizers is biological N fixation by cultivation of legumes (pulses, perennial legume based leys, or green manure in crop rotations) [51]. In the context of Bhutan, experiments have shown that green manuring is an option to achieve recommended nutrient levels in the soil for the cultivation of rice (but not for wheat) [35]. This potential, however, appears to be underutilized in Bhutan. Green manuring is rarely practiced [11] and pulses represent only about 3% of total cultivated area. Moreover, leguminous fodder crops are not yet used in Bhutan since most cattle husbandry systems are based on extensive grazing on pastures and in forests. In addition to legumes, further organic fertilizers such as leaf litter from forests and recycled materials from crop and household waste also play a rather minor role. Given the limited adoption of improved soil fertility management practices at the current stage and due to the lack of data, our model does not explicitly incorporate crop rotations and only includes manure as an organic fertilizer. Since multiple cropping and crop rotations are inherent features in the cropping system of both organic and conventional farmers in Bhutan [52], there is substantial potential to improve the adoption of N-fixing crops in order to lower the yield gap [53]. However, modelling their potential without sufficient data and recognizable efforts or initiatives undertaken to improve their adoption remains speculative.

OA is generally known to be more labour intensive than CA, but this factor receives little attention in the literature. The labour intensity of organic rice production in Bhutan, for instance, is 11% higher for organic farmers due to higher labour requirement for weeding and applying farmyard manure [28]. Using the novel approach to model field operations, we explicitly depict the technological trade-off between both systems and show that aggregate labour absorbed by crop activities increases by 2.3%. Unlike claims in literature ([54, 55], our results do not show that the higher labour intensity of OA results in positive welfare effects for agricultural households, because of higher income from agricultural labour. Following the 100% OA policy, wages only increase slightly in those regions with low conventional production. In AEZ3, where the OA conversion is most challenging, agricultural output drops significantly and cultivated land declines by -3.4%, resulting in a lower demand for labour by cropping activities. Furthermore, in AEZ2 the benefit of slightly higher agricultural wages is offset by the loss in purchasing power, due to higher food prices.

Since the OA conversion leads to higher labour requirement within specific periods, e.g., the periods of weeding and manuring, the depiction of seasonal labour would be intriguing and could influence changes in wages and land allocation. Yet, research on how to depict seasonality of labour markets in economy-wide models only started recently [56]. A further model limitation is the lack of labour mobility between agricultural and non-agricultural sectors. Non-agricultural wages are reduced at higher rates than agricultural wages, which implies that allowing migration between agriculture and non-agriculture sectors would result in an even higher decline of agricultural wages.

Our model framework does not incorporate the environmental benefits from OA, due to a lack of data and research, which would help to derive their potential magnitude. The mentioned leakage-effect would also require to also considerations with regard to the adverse effects on countries from which additional conventionally produced food is imported. Moreover, our model does not account for any possible spillover effects that might improve Bhutan’s brand as a high-value tourist destination. However, according to the model results, tourist arrivals would need to more than double, if the societal cost of converting to organic farming should be born from an increase in tourist royalties.

### Sensitivity analysis

In order to check the robustness of our model and results for variations in the yield gap, we simulated two further scenarios by varying the yield gap of all crops by +20% and -20%. Like in the reference scenario, this variation only affected those yield ratios that were found to be statistically significant.

**Fig 5:**
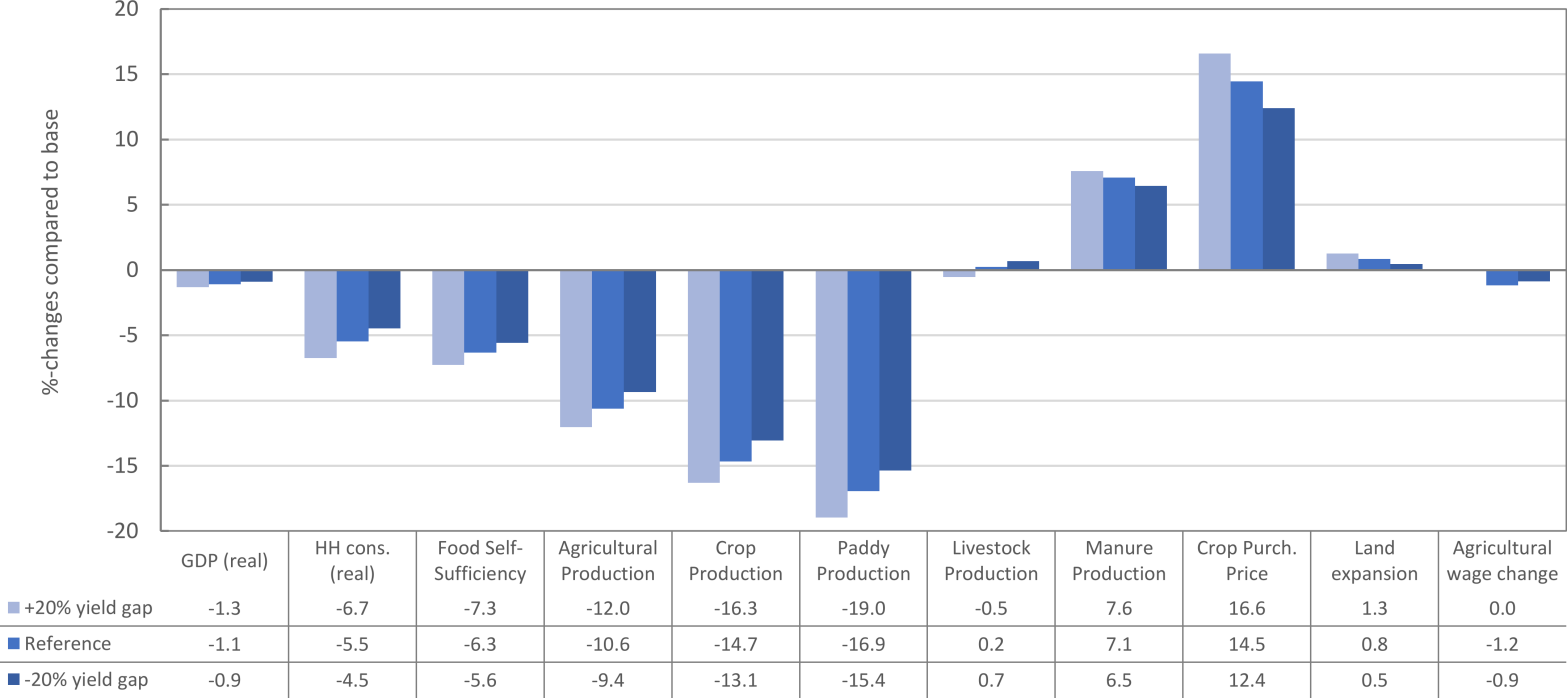
Sensitivity analysis to check robustness of main indicators for variation in the yield gap.

Following the described variation in yield gaps, real GDP varied only moderately, between -1.3% and 0.9% (Fig 5). The 20% increase in the yield gap triggered higher relative reductions in household consumption, food self-sufficiency, and agricultural production compared to a 20% decrease in the yield gap. This is due to the limited adaptive capacity of the agricultural sector to absorb a large reduction in its productivity. Manure production, for instance, cannot be increased infinitely and increases at a diminishing rate vis-à-vis the change in yield gap. In contrast, land cultivation increases (decreases) with a higher (lower) yield gap. Indeed, running an additional scenario without a yield gap, we even observe a drop in total land cultivation of -1.0%. Since agricultural labour and land are demanded in fixed shares, agricultural wages increase as more land is brought under cultivation.

### Policy implications

There is an obvious and serious conflict between the 100% OA policy with increased food self-sufficiency. In this paper, we assess this conflict in case of the current governmental approach of defining OA as “free of agrochemicals” while other aspects of OA being a holistic system are neglected [57]. Our study does not impose any adoption of improved organic farming practices, which have been proven to potentially close the yield gap in comparable situations [40], since there are no interventions or trends recognizable in Bhutanese agriculture that would make such an assumption reasonable. In case of an optimised organic farming system, the conflict between food self-sufficiency and OA may be reduced and it is an obvious policy implication, that this would require an adequate political strategy for the improvement of productivity of OA in Bhutan.

In order to increase yield levels of today’s organic by default farming system, government investments in research and extension are necessary. Yet, up to now research on organic farming is concentrated at one of the five national Renewable Natural Resources (RNR) research centres [16] and the research policy formulated in 2011 only refers to organic farming once in 24 pages by stating to “give primacy to organically clean agriculture and minimize the role of external inputs” [58]. Given the challenge of converting to 100% organic, the current research capacity is still too limited and would need to be expanded significantly [19]. Specifically, technology development (e.g., optimised fertilization strategies based on biological N fixation, development of plant-based pesticides, adaptation of crop rotations, technological solutions for labour intensive practices such as weeding in rice, breeding of adapted crop varieties, improvement of mixed farming systems with animal husbandry) for an intensification of the traditional agricultural systems is needed to improve yields in OA.

Besides research efforts and short-term dissemination of best-practice examples (peer-to-peer extension), demonstration farms and intensive training of extension workers could reduce the societal conversion costs. Access to export markets for high-value niche products might be another way to capitalize on the advantages of OA. Such opportunities could also arise in India in the long-run, where the awareness for quality and other added values of food products is growing among the middle class. However, the most important factor in the context of food self-sufficiency is the emergence of a market for organic produce inside Bhutan, which requires increased efforts to create consumer awareness.

Other political strategies of dealing with the conflict of food self-sufficiency and OA are less powerful. For example, excluding the paddy sector to achieve higher rice self-sufficiency would largely diminish the benefits of an organic conversion and entail the risk of agrochemicals leaking into other cropping activities. Alternative to banning agrochemicals, taxing agrochemical inputs would reduce their usage where they are least efficient, while still allowing them where they provide a high benefit-cost ratio. In Bhutan, levels of agrochemical use are low, which can imply high marginal-benefits while causing low marginal-damage to the environment. Finally, such alternatives that aim at less than 100% OA will lower the potential of branding Bhutan as a genuine “organic and green country”, which may be considered the most important benefit of OA in Bhutan.

Our results show the importance of increased conventional food imports to buffer the negative productivity shock on Bhutan’s cropping sector. This effect can hardly be avoided as any trade policy has to comply with Bhutan’s free trade agreement with India. A non-discriminatory measure would be to tax all agricultural goods that were not produced under organic standards, hence resulting in an effective import tax on most food items. However, this would further increase agricultural prices. A similar option would be to introduce sales taxes on agricultural produce, which could be rebated as production subsidies. This would lower the relative price difference between imported and local produce, but simultaneously provoke the risk of consumer protests in urban areas. As 100% organic is likely to benefit the tourism sector, policymakers could alternatively increase the tourism royalty in order to compensate farmers for lower productivity. This would benefit both farmers and consumers, yet the tourist’s willingness to accept such an increase in royalty is uncertain.

## Conclusions

We studied Bhutan’s policy to convert 100% to OA analysing the economy-wide effects of such an unprecedented large-scale conversion policy. From a method development perspective, we conclude that shifting land between organic and non-organic farming activities in a CGE with a detailed depiction of the agricultural production structure, including the specification of complementary and substitutable field operations, is an adequate method to analyse the economy-wide effects of a conversion from conventional to organic agriculture.

Regarding the implications of OA in Bhutan, we found a substantial yield gap between OA and CA for most crops despite the country’s low reliance on agrochemical inputs. We were able to show that lower yields and higher labour requirements in OA to substitute the previous use of pesticides and fertilizers in CA resulted in a strong contraction of agricultural output, substantial losses in welfare and negative implications for food security. Based on our data, converting Bhutan’s CA to the current organic by default farming system seems to be insufficient at fulfilling the promise of a 100% OA as a sustainable and environmentally friendly farming system that maintains current levels of food production. To reach this aim, further adaptations of cropping systems (fertilisation management, crop protection, integration of livestock) are necessary to narrow the current yield gaps and to develop truly holistic organic farming systems. We acknowledge that our database is far from sufficient to come up with conclusions regarding the potential of an optimized OA system, as little or no data for improved organic farming practices from field trials or farm pair comparisons exist for Bhutan. As for our model analysis, further research is needed to adequately address challenges that go beyond the scope of this study, such as potential environmental benefits and positive spillover effects, e.g., a boost in tourist arrivals.

Against the background of substantial efforts by the Bhutanese government to make agriculture more productive and commercial, our results showed that the 100% organic policy affects the cropping sector like a production tax without the opportunity of redistributing any tax income. Bhutan’s policymakers should recognize these trade-offs, prioritize accordingly, and develop strategies to mitigate the conflict of objectives between OA and high food self-sufficiency. Increased efforts in the area of agricultural research, extension outreach and market development are necessary in order to ensure that a 100% OA policy can be reconciled with an improvement of rural livelihoods. If these efforts result in the adoption of advanced organic farming practices and access to price premiums then Bhutan’s 100% conversion to OA may result in lower economic cost than assessed in this study and farmers may even benefit from conversion to improved OA.

## Supporting information

S1 Table Accounts of adopted 2012 Social Accounting Matrix. (PDF)

S1 Fig Use of agrochemicals in Bhutan 2005–2014. (PDF)

S2 Fig Percentage changes in livestock output, supply and purchaser prices after simulating a 100% organic policy. (PDF)

S1 Text Model extensions and parameters. (PDF)
