# Supplementary material for: Is Bhutan destined for 100% organic? Assessing the economy-wide effects of a large-scale conversion policy
Source: PLoS One. 2018 Jun 13;13(6):e0199025. doi: 10.1371/journal.pone.0199025 (PMC5999226; doi:10.1371/journal.pone.0199025)
Supplement: S1 Table — (PDF) [file pone.0199025.s001.pdf]

S1 Table. Accounts of adopted 2012 Social Accounting Matrix

Commodities

**Commodity Name**	**Description**
**Crops**	
Paddy	Cultivation of paddy rice on irrigated-land
Maize	
Other cereals	
Crop residues	By-product of paddy, maize and other cereals activities; disaggregated according to AEZ
Crop fodder	By-product of all crop producing activities; disaggregated according to AEZ
Vegetables	
Potato	
Spices	
Fruits	
**Livestock**	
Milk	
Beef	
Manure	Disaggregated according to AEZ
Live animals	
Draught power	Disaggregated according to AEZ
Other animal products	
**Field operations**	
Mechanical land preparation	Disaggregated according to AEZ
Draught-power land preparation	Disaggregated according to AEZ
Manual land preparation	Disaggregated according to AEZ
Organic fertilization	Disaggregated according to AEZ
Chemical fertilization	Disaggregated according to AEZ
Organic plant protection	Disaggregated according to AEZ
Chemical plant protection	Disaggregated according to AEZ
Other operations	Disaggregated according to AEZ
Compound feeding	Disaggregated according to AEZ
**Food products**	
Milled Rice	Milled paddy
Flour	Milled other cereals
Dairy	
Animal feed	
Tabaco and alcoholic beverages	
Ara	Home-brewed alcohol
Beverages	
**Forest products**	
Firewood	
Logs	
Non-wood forest products	
**Industrial products**	
Other minerals	
Other food	
Clothes	
Other manufacturing	
Fuels	
Chemical fertilizer	
Pesticides	
Electricity	

Services	
Construction services	
Transportation services	
Trade	
Lodging	
Other services	
Public services	

Activities

**Activity Name**	**Description**
**Crops**	
Paddy*	Disaggregated by AEZ and cultivation system (conventional versus organic)
Maize*	
Other cereals*	
Vegetables*	
Potato*	
Spices*	
Fruits*	
**Livestock**	
Cattle husbandry*	Disaggregated according to AEZ
Other animal husbandry*	Disaggregated according to AEZ
**Field operations**	
Mechanical land preparation*	Disaggregated according to AEZ
Draught-power land preparation*	Disaggregated according to AEZ
Manual land preparation*	Disaggregated according to AEZ
Organic fertilization*	Disaggregated according to AEZ
Chemical fertilization*	Disaggregated according to AEZ
Organic plant protection*	Disaggregated according to AEZ
Chemical plant protection*	Disaggregated according to AEZ
Other operations*	Disaggregated according to AEZ
Compound feeding*	Disaggregated according to AEZ
**Food products**	
Rice milling*	Disaggregated according to AEZ
Cereal milling*	Disaggregated according to AEZ
Dairy*	Disaggregated according to AEZ
Cereal processing*	Disaggregated according to AEZ
Ara production*	Disaggregated according to AEZ
Other food production	Producing the commodities: other food, animal feed, tobacco and alcoholic beverages, beverages
**Forest products**	
Community forestry*	Disaggregated according to AEZ
Forestry	
**Industrial products**	
Mining	
Textiles	
Other manufacturing	
Electricity generation	
**Services**	
Construction	
Transportation	
Wholesale and Trade	
Hotels and restaurants	
Other services	

Public services	

* denotes farm activities which require family-farm and hired-farm labour

Factors

**Factor name**	**Description**
**Labour**	
Skilled labour	
Unskilled labour	
Family-farm labour	Labour supplied by the farm household; Disaggregated according to AEZs
Hired-farm labour	Labour supplied by landless households; Disaggregated according to AEZs
**Agricultural land**	
Pasture land	Disaggregated according to AEZs
Irrigated land	Disaggregated according to AEZs and cultivation system
Rainfed land	Disaggregated according to AEZs and cultivation system
Orchards	Disaggregated according to AEZs and cultivation system
**Capital**	
Other machinery	Non-incorporated capital mostly used by farm-households
Powertiller	Disaggregated according to AEZs
Cattle	Disaggregated according to AEZs
Other animals	Disaggregated according to AEZs
Private capital	Incorporated capital of private enterprises
Public capital	Incorporated capital of public enterprises

Institutions

**Institutions**	**Description**
**Enterprises**	
Private enterprises	
Public enterprises	
**Households**	
Urban skilled households	Households in urban areas with primarily income from skilled labour
Urban unskilled households	Households in urban areas with primarily income from unskilled labour
Rural skilled households	Households in rural areas with primarily income from skilled labour
Rural unskilled households	Households in rural areas with primarily income from unskilled labour
Capital dependent households	Households dependent on income from private capital
Transfer dependent households	Households dependent on transfers from domestic and foreign households
Farm household	Households in rural areas living from family farm labour and land; Disaggregated according to AEZs
Landless household	Households in rural areas with primarily income from hired-farm labour; Disaggregated according to AEZs
**Government and taxes**	
Government	
Import taxes	
Excise taxes	
Sales taxes	
Direct taxes	
Indirect taxes	
**Other institutions**	
Investment / savings	
Stock changes	
Rest of the world	All trade partners of Bhutan
