# Supplementary material for: Is Bhutan destined for 100% organic? Assessing the economy-wide effects of a large-scale conversion policy
Source: PLoS One. 2018 Jun 13;13(6):e0199025. doi: 10.1371/journal.pone.0199025 (PMC5999226; doi:10.1371/journal.pone.0199025)
Supplement: S1 Fig — (PDF) [file pone.0199025.s002.pdf]

S1 Fig. Use of agrochemicals in Bhutan 2005-2014

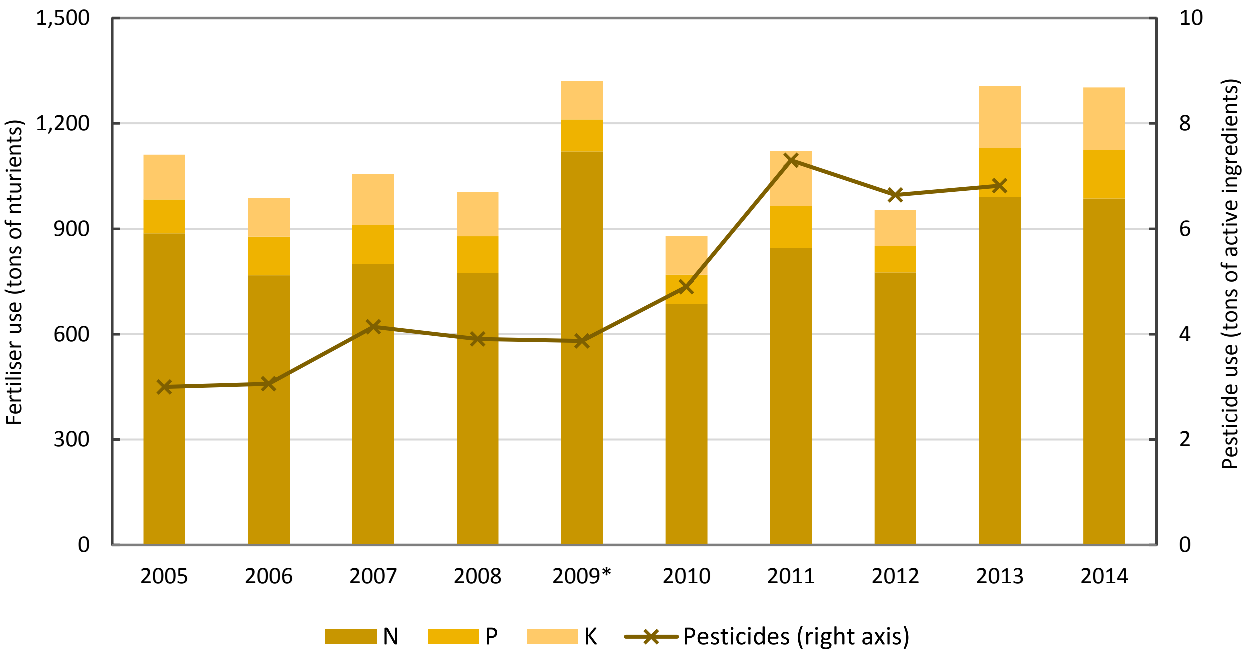

Data sources: Own calculation based on FAO (2017). *Fertilizer use data for 2009 was adjusted to the imported quantity of fertilisers as reported by official trade statistics (MoF, 2010).

References

FAO (2017), "FAOSTAT database", available at: http://www.fao.org/faostat/en/ (accessed 10 March 2017).

Ministry of Finance (MoF) (2010), *Bhutan Trade Statistics 2009*, Thimphu, Bhutan.
