# Supplementary material for: Is Bhutan destined for 100% organic? Assessing the economy-wide effects of a large-scale conversion policy
Source: PLoS One. 2018 Jun 13;13(6):e0199025. doi: 10.1371/journal.pone.0199025 (PMC5999226; doi:10.1371/journal.pone.0199025)
Supplement: S2 Fig — (PDF) [file pone.0199025.s003.pdf]

S2 Fig. Percentage changes in livestock output, supply, and purchaser prices after simulating a 100% organic policy.

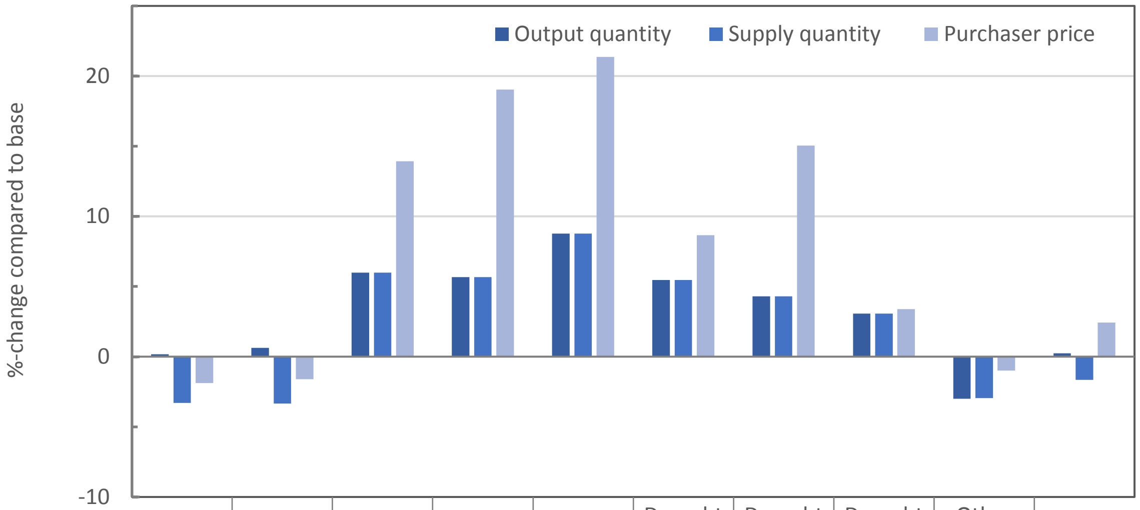

	Milk	Beef	Manure AEZ1	Manure AEZ2	Manure AEZ3	Draught power AEZ1	Draught power AEZ2	Draught power AEZ3	Other animal products	All lvst. products
Output quantity	0.2	0.6	6.0	5.7	8.8	5.5	4.3	3.1	-3.0	0.2
Supply quantity	-3.3	-3.3	6.0	5.7	8.8	5.5	4.3	3.1	-3.0	-1.6
Purchaser price	-1.9	-1.6	13.9	19.0	21.4	8.7	15.1	3.4	-1.0	2.4
