# Supplementary material for: Is Bhutan destined for 100% organic? Assessing the economy-wide effects of a large-scale conversion policy
Source: PLoS One. 2018 Jun 13;13(6):e0199025. doi: 10.1371/journal.pone.0199025 (PMC5999226; doi:10.1371/journal.pone.0199025)
Supplement: S1 Text — (PDF) [file pone.0199025.s004.pdf]

S1 Text. Model extensions and parameters

The original model structure of STAGE2 (McDonald and Thierfelder, 2015) was extended to fit the purpose of this study. The following three extensions were made:

Firstly, in addition to trade and transport margins for domestic consumption, the extended model also incorporates margins for exports, as due to the mountainous terrain in Bhutan considerable transport costs apply to export-goods as well.

Secondly, more flexibility regarding the output of selected multi-product activities (i.e. the livestock and forest activities) is allowed for by introducing a constant elasticities of transformation (CET) function as described by Punt (2013). This is particularly relevant for the production of animal products. For example, the cattle herding activity's outputs (milk, beef, manure, draught power and live animals) are not determined by fixed shares (Leontief) as in the standard model, but are made flexible to the degree of the CET parameter.

The third and most substantial extension was the adjustment of the production structure. The standard production structure of STAGE2 consists of a three-level nest aggregating intermediate inputs and production factors, which is adopted for the model's non-agricultural activities. The light shaded nests in Fig 1 represent this standard structure, with the exception that the land aggregate is shifted from level L3.1 to L5.2. Generally, we assume that intermediate inputs and value added components are aggregated according to fixed shares at L1. Intermediate inputs are also demanded in fixed shares (L2.1). Value added at L2.2 and factor aggregates below (L3.2 and 3.3) are aggregated using CES technology.

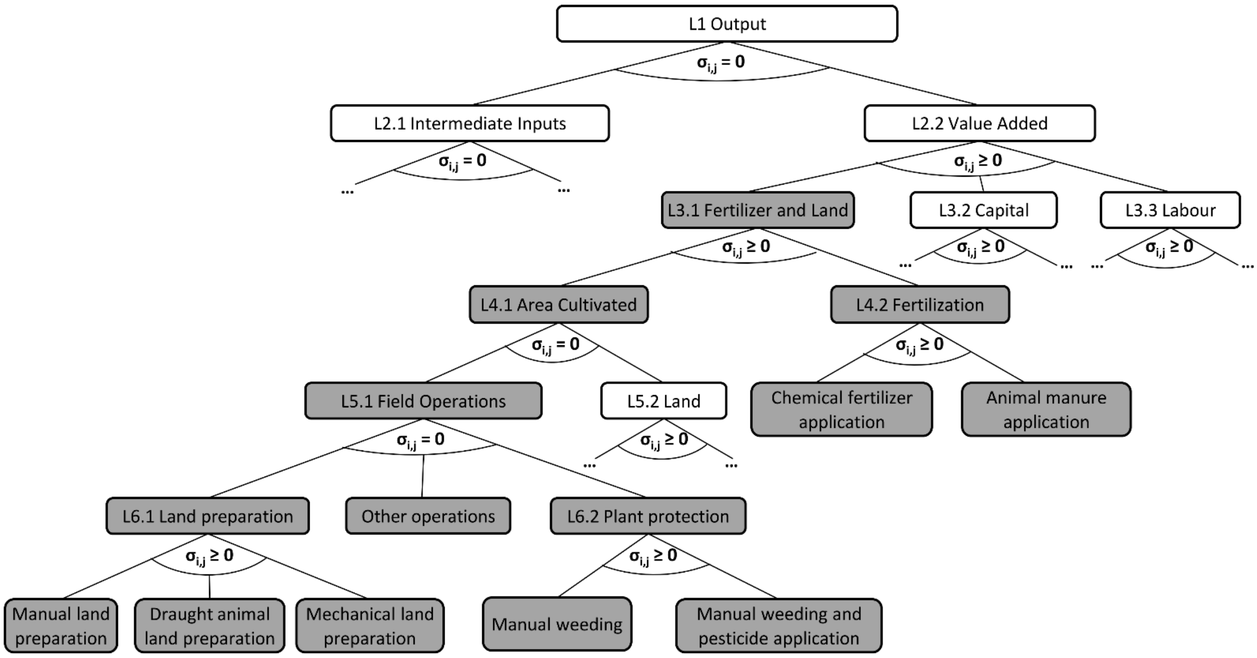

Fig 1 Extended production structure of economic activities. Sigmas denote substitution elasticities. Light shaded nests represent the standard three level structure and dark shaded nests represent the extended structure including field operations.

In case of agricultural activities, the production structure incorporates field operations, which are represented by the dark shaded nests in Fig 1. Since only cropping activities use field operations and land, this part remains empty for the remaining activities. Level L3.1 governs the activities' degree of intensification by aggregating area cultivated and fertilization. At this nest, we use a low substitution elasticity of 0.4 as proposed by (Bouët *et al.*, 2010) in the context of developing countries. An important relationship is captured at the Area Cultivated nest L4.1, which aggregates field operations and land in fixed shares. Assuming a fixed share at L4.1 makes land only substitutable with fertilizer at level L3.1, which is reasonable, as increasing the cultivated area also increases the requirement for labour for land preparation, harvesting, etc. At L4.2 the operations chemical fertilizer and animal manure application are aggregated, which includes the respective labour requirement. This is one exemplary technological trade-off represented in the structure, further trade-offs are the nests L6.1 (land preparation) and L6.2 (plant protection). These latter two are aggregated at fixed shares together with other operations

at L5.1. Other operations includes all field operations, which do not include any technological trade-off such as sowing, irrigation, and harvesting.[1]

For the value added nest L2.2 within this structure we apply elasticities from the GTAP database (Hertel *et al.*, 2008). The elasticity taken for the aggregation of capital (L3.2) and labour (L3.3) equals 1.5 and is based on Hertel (1997). For the elasticity of the land-fertilizer aggregate (L.31) we follow (Bouët *et al.*, 2010), which use an elasticity of 0.4 in the context of developing countries. No elasticities are needed at nest level 4.1 and 5.1, where the nest is aggregated using fixed shares assuming Leontief technology. This entails the logic that every unit of land requires the same bundle of field operations. The elasticity governing the aggregation of different land types is assumed to be 1.2, however, the choice of elasticity does not affect model results as at the current aggregation level all activities only use one type of land. The elasticity of aggregating chemical fertilizer and organic fertilizer at nest L4.2 is set as 1.2, which is within the range of estimates of 0.523 and 1.327 that Ali und Parikh (1992) found for non-tractorized and tractorized plots in Pakistan. The substitution of different land preparation technologies (mechanically, animal traction or manual) is assumed to be perfectly elastic, which is why we assume a very high elasticity of 6 at nest L6.1. At nest L6.2 the elasticity to aggregate plant protection operations is assumed to be 0.5, reflecting imperfect elasticity.

The Armington elasticities for the aggregation of imports and domestically produced goods are also taken from the GTAP database (Hertel *et al.*, 2008). Income elasticities for households are based on estimates provided by Minten and Dukpa (2010), which estimated an Almost Ideal Demand System (AIDS) for Bhutan using 2007 household data.

In case of livestock activities, labour enters at L5.1 and pasture land at L5.2. At L4.2, instead of fertilizers the different types of feed (Compound feed,crop residues and crop fodder) are aggregated.

References

Ali, F. and Parikh, A. (1992), “Relationships among labor, bullock, and tractor inputs in Pakistan agriculture”, *American Journal of Agricultural Economics*, Vol. 74 No. 2, pp. 371–377.

Bouët, A., Dimaranan, B.V. and Valin, H. (2010), *Modeling the global trade and environmental impacts of biofuel policies*, *IFPRI Discussion Paper*, Washington DC.

Hertel, T., McDougall, R., Narayanan, B. and Aguiar, A. (2008), *Chapter 14: Behavioral Parameters, GTAP 7 Data Base Documentation*, West Lafayette, USA.

Hertel, T.W. (1997), *Global Trade Analysis: Modeling and Applications*, Cambridge university press.

McDonald, S. and Thierfelder, K. (2015), “A Static Applied General Equilibrium Model: Technical Documentation. STAGE Version 2: January 2015”, *Model Documentation*.

Minten, B. and Dukpa, C. (2010), *An analysis of household food demand in Bhutan*, Ministry of Agriculture and Forests of Bhutan, Thimphu.

Punt, C. (2013), “Modelling multi-product industries in computable general equilibrium (CGE) models”, Stellenbosch: Stellenbosch University, 2013.
